# Stereotactic radiotherapy for lung cancer using a flattening filter free Clinac

**DOI:** 10.1120/jacmp.v10i1.2880

**Published:** 2009-01-27

**Authors:** Oleg N. Vassiliev, Stephen F. Kry, Joe Y. Chang, Peter A. Balter, Uwe Titt, Radhe Mohan

**Affiliations:** ^1^ Department of Radiation Physics The University of Texas M. D. Anderson Cancer Center Houston Texas U.S.A.; ^2^ Department Radiation Oncology The University of Texas M. D. Anderson Cancer Center Houston Texas U.S.A.

**Keywords:** lung cancer, flattening filter, clinical linear accelerator, stereotactic radiotherapy

## Abstract

The objective of this study was to assess the feasibility of stereotactic radiotherapy for early stage lung cancer using photon beams from a Varian Clinac accelerator operated without a flattening filter. Treatment plans were generated for 10 lung cancer patients with isolated lesions less than 3 cm in diameter. For each patient, two plans were generated, one with and one without the flattening filter. Plans were generated with Eclipse 8.0 (Varian Medical Systems) commissioned with beam data measured on a Clinac 21EX (Varian Medical Systems) operated with and without the flattening filter. Removal of the flattening filter increased the dose rate. The median beam‐on time per field was reduced from 25 sec (with the filter) to 11 sec (without the filter), increasing the feasibility of breath‐hold treatments and the efficiency of gated treatments. Differences in a dose heterogeneity index for the planning target volume between plans with flattened and unflattened beams were statistically insignificant. Differences in mean doses to organs at risk were small, typically about 10 cGy over the entire treatment. The study concludes that radiotherapy with unflattened beams is feasible and requires substantially less beam‐on time, facilitating breath‐hold and gating techniques.

PACS numbers: 87.56.bd, 87.53.Ly

## I. INTRODUCTION

Surgical resection is the preferred treatment for the early stage non‐small‐cell lung cancer. It results in high local control and survival rates. For patients who are not amenable to surgery, stereotactic radiotherapy (SRT) is an alternative method for the curative treatment. Several studies,^(^ ^1^ ^–^ ^20^ ^)^ including phase I and II clinical trials, have shown that this method is reasonably safe for medically inoperable patients who usually suffer from coexisting morbidities or general frailty. For such patients, the studies report overall favorable results of SRT in terms of local control and toxicity. In a cohort of medically operable patients receiving SRT survival rates “potentially equivalent to those of surgery” have been reported.^(8)^

The efficiency of SRT is adversely affected by respiratory motion. Several methods have been developed to improve the precision of dose delivery to the moving tumor and reducing irradiation of healthy lungs,^(21)^ but there are drawbacks to using them. For instance, respiratory gating increases treatment delivery time and breath‐hold techniques add patient discomfort. Increasing the dose rate of photon beams used for SRT can alleviate these problems by reducing beam‐on time. Previously, we reported on characteristics of photon beams from a clinical accelerator, the Clinac 21EX (Varian Medical Systems, Palo Alto, CA), which was operated with the flattening filter removed from the beamline.^(^ ^22^ ^–^ ^26^ ^)^ The most notable result of removing the filter was a large increase in the dose rate. Although the beam is not flat when the filter is absent, flatness is not necessary for intensity‐modulated radiotherapy (IMRT). We have demonstrated this in a treatment planning study for the prostate.^(27)^ Treatment planning for SRT of lung tumors, however, is different. Usually it is forward planned and does not involve intensity modulation. Therefore, it remains to be seen whether SRT with unflattened beams is feasible. The goals of this work were to determine the feasibility of developing clinically acceptable SRT treatment plans using unflattened beams, compare them with typical plans developed with flattened beams, and explore the potential benefits and drawbacks of using unflattened beams for this type of treatment.

## II. MATERIALS AND METHODS

Ten lung cancer patients with small, less than 3 cm diameter, isolated lesions were selected at random from the pool of patients undergoing SRT at our clinic. All patients were simulated using four‐dimensional computed tomography (4DCT) (GE Medical Systems, Milwaukie, WI) and were immobilized using a large vacuum formed custom immobilization device (Medical Intelligence, Schwabmünchen, Germany). These images were transferred to our treatment planning system. The envelope of the gross tumor volume (GTV) motion on all 10 phases of the 4DCT was determined by contouring the GTV on the maximum intensity projection image (MIP) and then reviewing it across all 10 phases and editing as necessary. This structure will be referred to as the iGTV. The Internal Target Volume (ITV) was created by expanding the iGTV by 7 mm and then editing this expansion based on clinical judgment of disease spread. The Planning Target Volume (PTV) was then created by expanding the ITV by 3 mm to account for daily setup errors. On each treatment day, patients are imaged in treatment position using a linac mounted cone beam CT system (OBI, Varian Associates, Palo Alto, CA). These daily images are compared to reference images and contours from our treatment planning system using in‐house software. The in‐house software reports couch shifts to bring the patient to the planned isocenter. Anterior‐posterior and lateral verification images are also acquired prior to treatment using a portal imager to ensure the couch shifts were made correctly and that the patient has not moved since the cone beam CT was acquired.

PTV size ranged from 29.2 to 149.3 cm^3^ with a median of 73.1 cm^3^. SRT was forward planned for all patients using Eclipse 8.0 treatment planning system (Varian Medical Systems, Palo Alto, CA). It was commissioned with beam data measured on a Clinac 21EX operated with and without the flattening filter. The measurements are reviewed elsewhere.^(23)^ To perform measurements without a flattening filter in the beam, the Clinac was used in service mode and several interlocks were overridden. The filters, for 6 MV and 18 MV beams, are mounted on a carousel. The carousel was rotated using manual controls so that the filter moved out of the beam and was replaced with a vacant slot. For each patient two plans were generated: one for administering dose with the flattening filter and one for administering the dose without the flattening filter. The prescription doses were: 4 fractions of 12.5 Gy per fraction, the standard for these treatments in our clinic, for 7 patients; 4 fractions of 10 Gy per fraction for 2 patients; and five fractions of 10 Gy per fraction for 1 patient. The dose was prescribed to an isodose line covering at least 95% of the PTV. The prescription was generally to 80%‐90% of the maximum dose to provide a shaper falloff outside of the treatment area. This also provided a boost dose to the GTV; however, this was not an explicit goal. Eight patients were treated with 6 MV beams and two patients with a combination of 6 MV and 18 MV beams. Beam modifying devices included wedges and/or a multileaf collimator. The number of fields per plan was 5–8. The treatment plans using flattened beams were those that were developed clinically. Plans that did not incorporate flattening filters were done for comparison only and were not used clinically. Each plan developed using unflattened beams involved beam angles and modifiers similar to those used in the plan employing flattened beams. Dose distributions were calculated using the pencil beam convolution algorithm. The calculation grid was 2.5 mm. The Batho power law inhomogeneity correction was applied. Dosimetric differences between flattened‐beam and unflattened‐beam treatment plans were determined from dose‐volume histograms. Additionally, the integral dose for normal tissue was calculated as the product of mean dose and volume for normal tissue, excluding the PTV. The integral dose for the ipsilateral lung was calculated similarly. Two‐tailed paired t‐tests were used to evaluate the significance of observed dosimetric differences.

## III. RESULTS AND DISCUSSION

### A. Dose distributions

**Figure 1:**
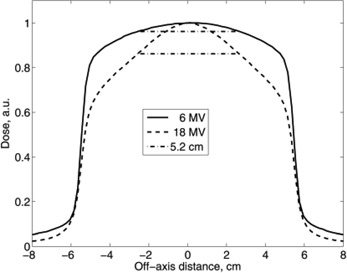
Cross‐field profiles of unflattened beams compared to the median PTV size of 5.2 cm. The solid line represents 6 MV photons, the dashed line 18 MV photons, and the dash/dot line represents the median PTV size of 5.2 cm.

Unflattened 6 MV beams are fairly flat within a few centimeters of the beam's central axis.^(23)^ At higher energies the beam is more forward‐peaked. Figure 1 shows dose profiles of 6 MV and 18 MV unflattened beams measured in a water phantom and the median size of the PTV as 5.2 cm. The ratio of maximum to minimum doses in these profiles, within that 5.2 cm, was 1.04 for the 6 MV beam and 1.16 for the 18 MV beam. Even though these ratios exceed the normally accepted beam flatness tolerance, one has to keep in mind that the thoracic region has a highly nonuniform density. The flattening filter is designed to achieve a flat dose distribution only at a certain depth, in a homogeneous phantom, for a beam incident at a right angle to the phantom's flat surface. In actual lung cancer treatments, inhomogeneities in the beam path strongly distort the profiles of beams reaching the PTV. Taking also into consideration the relatively small PTVs, it appears that flattened beams do not offer much advantage in lung SRT over unflattened beams. Nevertheless, it is important to determine whether acceptably uniform dose distributions in the PTV can be achieved with unflattened beams. To quantify dose uniformity in the PTV, we have calculated for each plan the heterogeneity index (HI). HI is a ratio of the doses delivered to 5% and 95% of the PTV.^(28)^ This parameter is reported in Table 1. These data indicate no significant differences between treatment plans with flattened beams and those using unflattened beams. Similarly, neither median V90 nor median V100 were significantly different, being higher in plans with unflattened beams, by 2.1 cm^3^ (p=0.9) and 0.6 cm^3^ (p=0.4), respectively.

**Table 1:** Dose heterogeneity index for the PTV defined as the ratio of the doses delivered to 5% and 95% of the PTV^(27)^. The p‐value is 0.3.

*Patient*	*1*	*2*	*3*	*4*	*5*	*6*	*7*	*8*	*9*	*10*
With filter	1.11	1.15	1.11	1.06	1.14	1.08	1.09	1.13	1.13	1.15
Without filter	1.12	1.14	1.12	1.06	1.12	1.09	1.08	1.12	1.18	1.17

**Figure 2:**
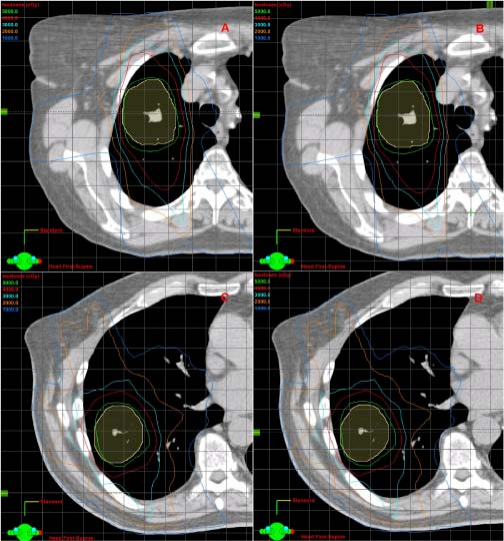
Planning CT images with isodose lines for two patients. Images A and B are from patient 1, C and D from patient 2. Images A and C show plans with flattened 6 MV beams while B and D show plans with unflattened 6 MV beams. Isodose lines represent planned doses of 50 Gy (green), 40 Gy (red), 30 Gy (cyan), 20 Gy (orange), 10 Gy (blue). The maximum dose in these plans is less then 60 Gy. The PTV is shown in yellow.

**Figure 3:**
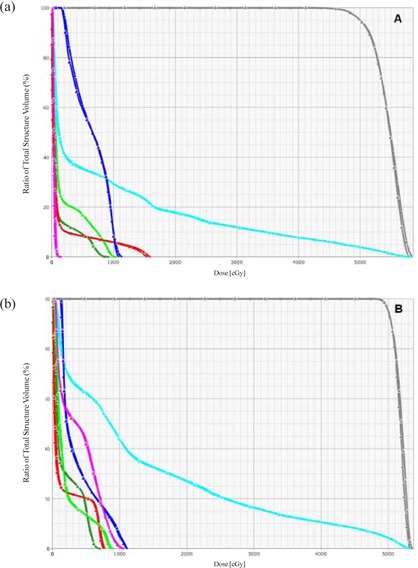
Dose volume histograms for patients 1 and 2, images A and B, respectively. Data for plans with flattened beams are indicated by squares. Data for plans with unflattened beams are indicated by triangles. Dark gray lines represent the PTV; cyan, ipsilateral lung; dark green, contralateral lung; light green, esophagus; red, spinal cord; dark blue, carina; magenta, the heart.

Figure 2 shows CT images with isodose lines for two patients. For each of the two patients two plans are shown: with flattened and unflattened beams. All the beams in Fig. 2 are 6 MV photons. Apart from having much higher dose rates, unflattened beams are similar to flattened beams for the small field sizes used in this study. Thus the plans developed with flattened and unflattened beams look very similar (Fig. 2). However, it is notable that the 10 Gy isodose (blue line) tends to be slightly closer to the surface in plans with unflattened beams than in plans with flattened beams. This is most visible in C and D of Fig. 2, near the skin. This trend is the result of unflattened beams having softer spectra and therefore a higher surface dose than flattened beams. The spectra, skin dose, and general depth dose data were reported in previous studies.^(^ ^22^ ^–^ ^24^ ^)^ This could be a potential problem for tumors located along the chest wall where skin dose becomes a limiting factor. To reduce the skin dose, the flattening filter free accelerator may need to operate at an energy that is greater than 6 MV, perhaps at 8 MV or 10 MV.^(29)^ The similarity of the dose‐volume histograms for these two patients (Fig. 3) confirms that differences between plans with flattened and unflattened beams are small.

However, an analysis of dose distributions for all ten patients revealed some differences in doses to organs at risk. Results of the analysis are summarized in Table 2. It shows mean and maximum doses to organs at risks: carina, cord, esophagus, heart and contralateral lung. Mean doses to all the listed organs, except the carina, tend to be higher in plans with unflattened beams, although the difference in dose is quite small. For these organs, the mean dose was calculated for each of the 10 patients. The median value of these mean organ doses is higher by 14–19 cGy in plans with unflattened beams. This difference is generally too small to be visualized on DVHs (Fig. 3) and is unlikely to be clinically significant. For the cord, heart and contralateral lung, these differences appear to be statistically significant. A review of treatment plans revealed that the difference is larger in those plans where at least one beam passes through part of an organ at risk before it reaches the PTV. Then, in these plans, the differences can be explained by the properties of depth‐dose dependencies. More specifically, beyond the depth of the maximum dose, the dose decreases with depth faster in unflattened beams then it does in flattened beams.^(23)^ This problem may be overcome by increasing the beam energy. Previous studies have shown that depth‐dose dependence similar to that of a flattened 6 MV beam can be achieved in an unflattened beam by increasing its energy from 6 MV to 8 M V.^(29)^ Maximum doses to organs at risk reported in Table 2 do not differ significantly between plans with flattened beams and those with unflattened beams. To some extent, this result can be attributed to the larger statistical fluctuations in maximum dose than in mean dose.

**Table 2:** Mean and maximum doses to organs at risk. Sample median values.

*Organ*	*Mean dose, Gy*		*p‐ value*	*Maximum dose, Gy*		*p‐ value*
	*With filter*	*Without filter*		*With filter*	*Without filter*	
Carina	5.07	5.03	0.4	11.3 11.1	0.3
Cord	1.43	1.57	0.007	7.36 7.65	0.5
Esophagus	2.20	2.35	0.06	12.4 12.6	0.5
Heart	0.81	1.00	0.001	11.5 9.82	0.4
Lung, contralateral	1.35	1.54	0.03	13.9 14.4	0.4

**Table 3:** Normal tissue integral doses for the whole body and ipsilateral lung. Sample median values, Gy×liter.

	*With filter*	*Without filter*	*p‐value*
Whole body	55.0	57.2	<0.001
Lung, ipsilateral	15.2	15.2	0.4

Another quantity of interest is the normal tissue integral dose (NTID) for the whole body and for the ipsilateral lung (see Table 3). The NTID for the whole body is higher in plans with unflattened beams. This result is consistent with findings from a previous study of prostate IMRT with unflattened beams.^(27)^ The main reason for a higher whole‐body NTID is a softer photon spectrum of unflattened beams, which causes a higher dose in the build up region near the surface where the beam enters the body. Beyond the build up region, closer to the PTV, dose distributions are more similar and the NTID for the ipsilateral lung is the same for plans with flattened beams and plans with unflattened beams.

For the target volume and abutting structures, the unflattened beams offered comparable dosimetric coverage as compared to flattened beams. The unflattened beams also offer reduced secondary dose to the patient at greater distances from the treatment field, through reduced head leakage and collimator scatter and less neutron production for the high‐energy beams.^(^ ^23^ ^–^ ^25^ ^)^ Reduced secondary dose reduces the potential for late effects.

### B. Beam‐on time

Removal of the flattening filter results in a large increase in the dose rate.^(23)^ To estimate the impact of the dose rate increase on treatment delivery time we have calculated beam‐on time for every field in the treatment plans we have developed in this study. The data were calculated assuming that the treatments were delivered at 600 monitor units per minute with the filter, and that the current of electrons incident on the bremsstrahlung target was the same with and without the filter. With the filter removed, the total beam‐on time was reduced on average by a factor of 2.31 (±0.02 standard deviation) for plans in which only 6 MV beams were used. In the two patients for whom a combination of 6 MV and 18 MV beams was used, the time reduction factors were 2.8 and 3.0. The median beam‐on time per field was reduced from 25 sec with the filter to 11 sec without the filter. Hanley et al.^(30)^ have reported “a comfortable breath‐hold duration of 12–16 sec” for patients undergoing radiation therapy for lung tumors. Thus, our data suggest that using unflattened beams for lung SRT improves the feasibility of the breath‐hold gated delivery technique. The Trilogy Stereotactic System from Varian Medical Systems can deliver treatments at 1000 MU/min (i.e. faster by a factor of approximately 1.7 than a standard Clinac operated at 600 MU/min). This is a lesser improvement than the factor of 2.3 to 3.0 reported above for the flattening filter free Clinac. Furthermore, the enhanced dose rate with the Trilogy machine requires a field size no larger than $15\times 15\;{\text{cm}}^{2}$, whereas the flattening filter free Clinac has no such limitation.

## V. CONCLUSIONS

Dose distributions in treatment plans for lung SRT with unflattened beams were close to those achieved in treatment plans with flattened beams. However, delivery of dose in unflattened beams required substantially less beam‐on time, making breath‐hold gated SRT treatments for lung tumors much more feasible with this technique than with SRT in which the flattening filter is present. Reduced beam‐on time also helps improve the efficiency of respiratory gated treatments.

## ACKNOWLEDGEMENTS

This work was partially supported by Varian Medical systems with a research grant.
